# Acute thrombocytopenia and fibrinogen reduction occurring after nintedanib treatment for immune checkpoint inhibitor-related pneumonia: a case report

**DOI:** 10.3389/fonc.2025.1561440

**Published:** 2025-08-07

**Authors:** Jian Zheng, Nan Zhou, Dexiang Pang

**Affiliations:** ^1^ Oncology Department, The Second Affiliated Hospital of Zhejiang Chinese Medicine University, Hangzhou, Zhejiang, China; ^2^ Oncology Department, The Traditional Chinese Medicine Hospital of Linping District, Hangzhou, Zhejiang, China

**Keywords:** drug-induced thrombocytopenia, fibrinogens abnormal, checkpoint inhibitor pneumonitis, nintedanib, case report

## Abstract

**Background:**

Nintedanib, a small molecule multi-target tyrosine kinase inhibitor, can block the fibrosis process and slow disease progression. Acute thrombocytopenia and fibrinogen reduction caused by nintedanib is a rare clinical event, with few studies reported.

**Case presentation:**

We report the case of an 89-year-old male diagnosed with advanced renal cell carcinoma who developed immune-related interstitial lung disease after treatment with sintilimab injection combined with axitinib. After prescribed nintedanib treatment, the patient’s platelet count decreased from 241×10 μ g/L to 49×10 μ g/L and fibrinogen decreased from 5.61 g/L to 0.76 g/L. Based on the patient’s medical history, laboratory test results, and examination results, the diagnosis was made that it was nintedanib-induced reduction in platelet and fibrinogen levels. After discontinuation of nintedanib, the patient’s platelets and fibrinogen returned to normal, and no further reduction in platelets and fibrinogen was observed during the follow-up period. This case report suggests to physicians that if there is an unexplained decrease in platelet and fibrinogen levels during nintedanib treatment, nintedanib-induced factors should be considered.

**Conclusion:**

Thrombocytopenia and fibrinogens abnormal are rare but serious adverse effect of nintedanib. This case highlights the importance of early recognition and monitoring of platelet counts and coagulation function in patients receiving nintedanib. Suspect drug discontinuation and active supportive care are essential. Further research is needed to elucidate the underlying mechanisms and then make corresponding treatment recommendations.

## Introduction

Immune checkpoint inhibitor-related pneumonia is a form of lung injury linked to immune checkpoint inhibitors (ICIs) that presents with a range of clinical symptoms, imaging findings, and pathological features. The incidence of this condition in real-world settings is notably higher than that observed in clinical trials, estimated at 3-5%. A systematic review and meta-analysis indicated that the incidence rates for grade I-V and grade III-V CIP were 0.16 (95% CI: 0.14-0.18) and 0.06 (95% CI: 0.05-0.08) (1), respectively, with more than 25% of patients experiencing mortality during ongoing ICI-P treatment (2). This condition is primarily marked by pulmonary inflammation, and treatment typically involves glucocorticoids and oxygen therapy, along with anti-infection measures when necessary. However, excessive T lymphocyte activation and the proliferation of fibrotic factors can lead to the development and worsening of pulmonary fibrosis, for which there is currently no established treatment protocol. Nintedanib, a multi-targeted tyrosine kinase inhibitor, demonstrates anti-fibrotic properties by inhibiting the release of inflammatory factors, fibroblast activity, and extracellular matrix deposition. It has been utilized in treating idiopathic pulmonary fibrosis and other interstitial lung diseases, making it a potential therapeutic option for immune checkpoint inhibitor-related pneumonia. In the INPULSIS analysis report, the main adverse reaction of nintedanib was diarrhea (3), whereas thrombocytopenia (4, 5) and fibrinogen concentration decreased were rare adverse reactions.

This report details a case of a patient who experienced acute thrombocytopenia and reduced fibrinogen levels while being treated with nintedanib for pneumonia caused by immune checkpoint inhibitors. Our goal is to raise awareness among clinical practitioners and pharmacists within the multidisciplinary team. This awareness is essential for enabling quick identification, effective management, and consistent application of the medication.

## Case report

**Figure 1:**
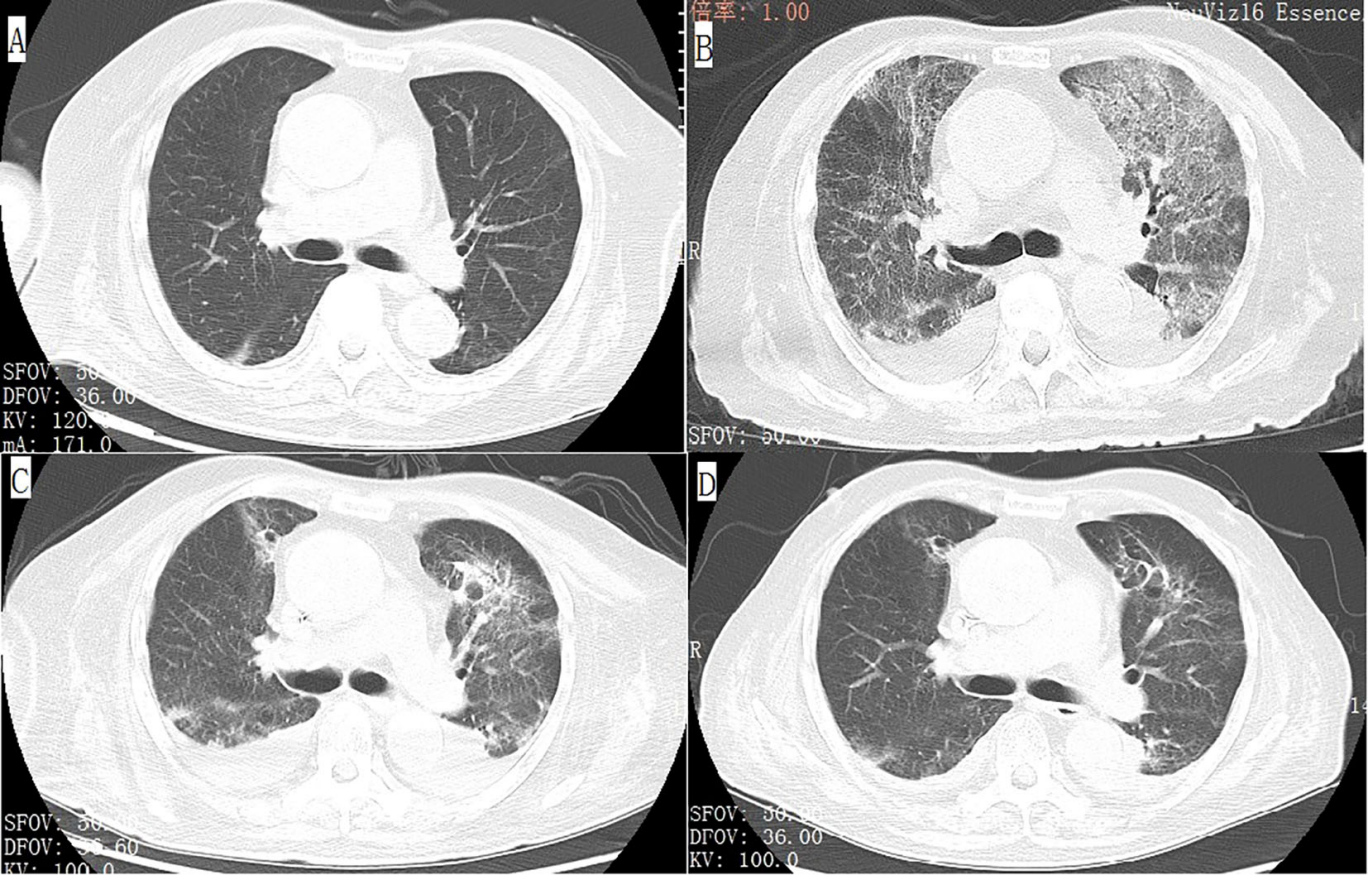
Axial view of chest computed tomography (CT) demonstrates that the patient’s baseline examination is unremarkable **(A)**, but after the onset of the disease, there is interstitial inflammation in both lungs, bilateral pleural effusion accompanied by adjacent lung tissue atelectasis **(B)**. After treatment, these lesions showed significant improvement **(C, D)**.

**Figure 2:**
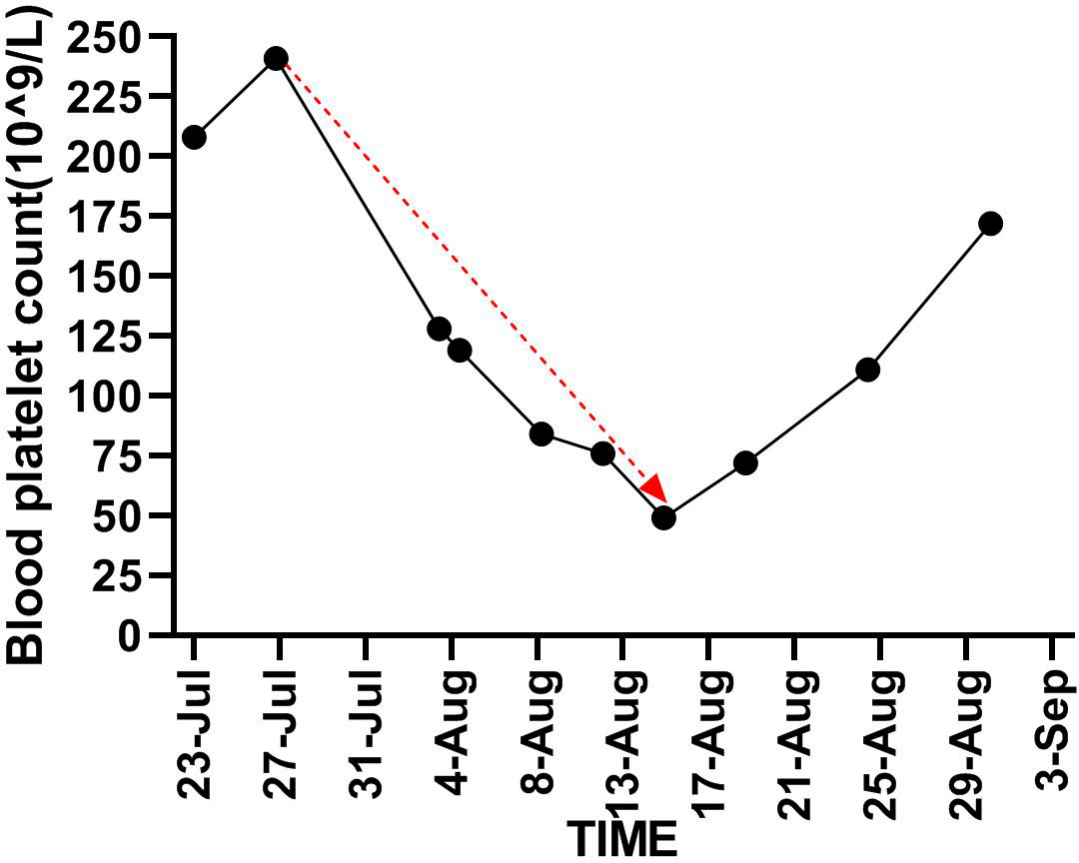
Demonstrates trend in platelet counts from the time of initiation of Nintedanib until it is stopped (red line).

**Figure 3:**
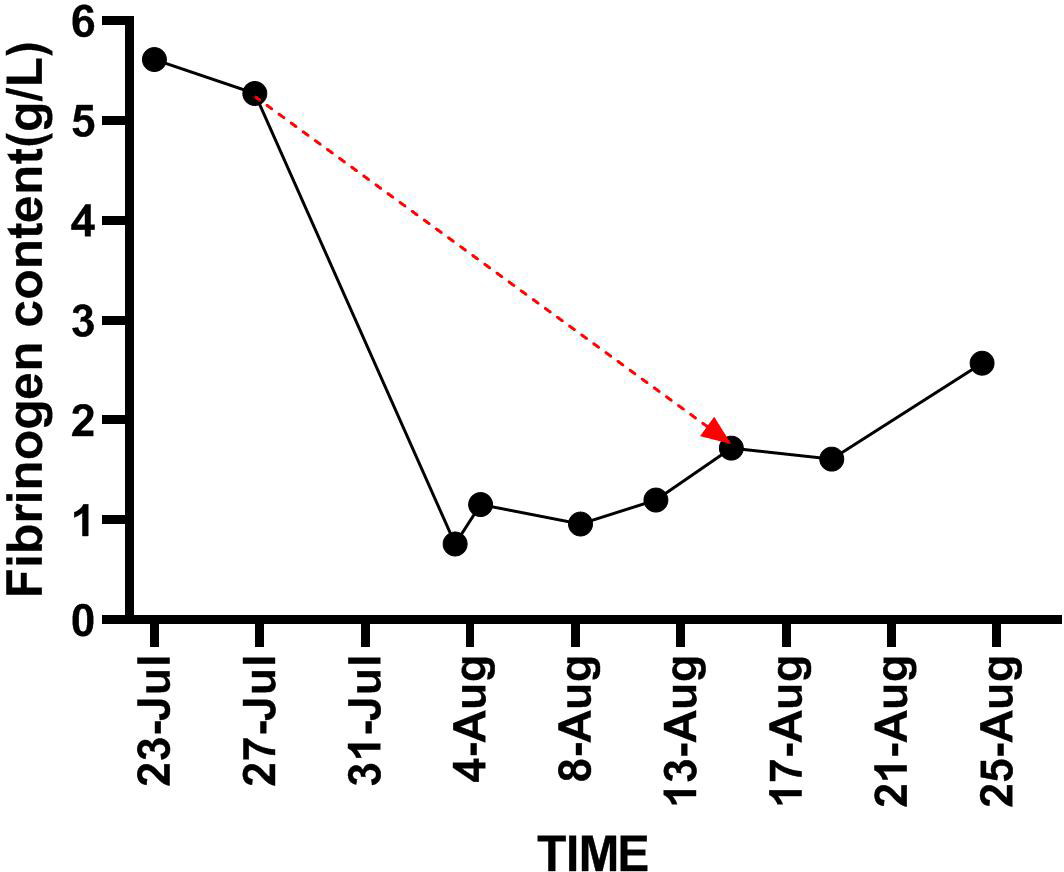
Demonstrates trend in fibrinogen content from the time of initiation of nintedanib until it is stopped (red line).

An 89-year-old male patient with a medical history of chronic renal insufficiency, hypertension, renal cysts, gouty arthritis, and gallstone disease was on a long-term medication regimen that included benazepril, α-keto acid, bailing capsules, irbesartan, and amlodipine. He was diagnosed with left kidney cancer over a year ago and underwent treatment with sintilimab combined with axitinib, with the last treatment occurring on April 18, 2022. Following this treatment, he experienced grade 3 hypertension and bullous dermatitis, leading to the discontinuation of these medications. Regular follow-ups have indicated a stable tumor status. The patient has no history of smoking and consumes alcohol moderately (**Figure 1A**). On July 23, 2022, he visited the hospital after experiencing a week-long episode of fever and shortness of breath. The fever, which reached a maximum temperature of 38.6°C, began without any obvious cause. Alongside the fever, he reported significant shortness of breath, a mild cough, and limited sputum production. A local diagnosis suggested pneumonia, prompting the initiation of empirical treatment with meropenem; however, his clinical symptoms did not improve. Upon transfer to our hospital on July 23, he presented with a body temperature of 38°C, a heart rate of 101 beats per minute, a respiratory rate of 22 breaths per minute, and blood oxygen saturation at 90%. A physical examination revealed scattered coin-sized vesicles across his body, with some lesions having scabbed over. Auscultation of the lungs indicated coarse breath sounds bilaterally, along with clear moist rales and wheezing sounds in the left lung. Laboratory tests conducted upon admission showed normal platelet (208×10^9/L) and white blood cell (5.8×10^9/L) counts, but the hemoglobin level was low at 84 g/L. C-reactive protein was elevated at 93.38 mg/L, while procalcitonin was measured at 0.24 ng/ml. Tests for Influenza A, Influenza B, Respiratory Syncytial Virus (RSV), Adenovirus, and the novel coronavirus (2019-nCoV) returned negative results. The arterial blood gas analysis revealed a PO2(T) of 47.6 mmHg, a sO2 of 93%, and a pO2/FiO2 ratio of 116 mmHg. Biochemical tests indicated elevated levels of creatinine at 113.2 μmol/L and lactate dehydrogenase (LDH) at 317 U/L, both exceeding the normal range. However, the results for electrolytes, liver function tests, thyroid-stimulating hormone, and cortisol levels were within normal limits, as were prothrombin time and activated partial thromboplastin time. Notably, D-dimer levels were elevated at 5.83 mg/L, and fibrinogen (FIB) was also higher than normal at 5.61 g/L. A chest CT scan showed interstitial inflammation in both lungs, along with pleural effusion on both sides and adjacent lung tissue insufflation (**Figure 1B**). No identifiable pathological microorganisms were detected in sputum and blood cultures. In response to these findings, immediate treatment was initiated, including oxygen therapy, methylprednisolone injection, meropenem powder injection, ambroxol, and other supportive measures. By July 27, 2022, there was a significant improvement in symptoms of fever and dyspnea, and laboratory tests indicated a marked decrease in CRP levels to 17.60 mg/L compared to previous results. The platelet count was recorded at 241×10^9/L. The liver was consistent, kidney function was improved, and creatinine values were within normal ranges. Subsequently, treatment with “Nidanib 100 mg bid” was initiated. On August 1, the patient exhibited subcutaneous bruising on the left upper limb, which progressively worsened. Laboratory tests revealed a platelet count of 128×10^9/L, while the C-reactive protein (CRP) level had decreased to 2.2 mg/L, falling within the normal range. However, the fibrinogen (FIB) detection value was notably low at 0.76 g/L, significantly lower than prior results. Following an infusion of human fibrinogen, the level increased to 0.96 g/L. By August 9, the platelet count had dropped to 84×10^9/L, falling below the normal threshold, and there was no observed improvement in the bruising of the upper limb. The Naranjo Scale score is not less than 5 points. On August 15, the platelet count further declined to 49×10^9/L, prompting the cessation of Nidanib treatment. Subsequently, the subcutaneous bleeding in the patient’s left upper limb began to improve. By August 25, a follow-up test indicated that the platelet count had risen to 111×10^9/L (**Figure 2**), and the fibrinogen level was recorded at 2.57 g/L (**Figure 3**). Further follow-up assessments demonstrated that both the platelet and fibrinogen levels were within normal limits, and there was a noted reduction in interstitial inflammation in both lungs (**Figures 1C, D**), with the evaluation of renal tumors remaining stable.

## Discussion

With the advancement of precision medicine, new anti-tumor drugs, particularly immune checkpoint inhibitors (ICIs), have greatly enhanced the effectiveness of comprehensive cancer treatment. However, the management of immune-related adverse events (irAEs) has become increasingly crucial. Among these, immune checkpoint inhibitor-related pneumonia, also known as checkpoint inhibitor pneumonitis (CIP), stands out as the most common and potentially fatal irAE, responsible for 35% of deaths associated with programmed cell death protein 1 (PD-1) and its ligand (PD-L1) (6). The clinical manifestations, imaging findings, and pathological characteristics of CIP can vary, with interstitial pneumonia being one of its forms (7). Research indicates that an increase in specific immune cell populations, such as CD57+CD8+ T cells (which also express TIGIT, LAG3, TIM-3, and PD-1), FCRL5+ B cells, and CCR2+CCR5+CD14+ monocytes that express immune checkpoints, may contribute to the development of ICI-related interstitial pneumonia (8). Prompt and appropriate use of glucocorticoids has been shown to improve lung function in affected patients, while in more severe cases, immunosuppressants may be necessary. Additionally, antifibrotic agents like nintedanib and pirfenidone are being utilized in treatment strategies.

This case centers on a patient with inoperable renal cell carcinoma who faced severe grade 3-4 toxic reactions following treatment with a standard multi-target inhibitor in combination with immune checkpoint inhibitors (ICIs), which ultimately led to the discontinuation of anti-tumor therapy. Interstitial pneumonia developed in the 31st week after the initiation of treatment and 13 weeks after the therapy was halted. A comprehensive series of pathogen and immunological tests were performed, including screening for 2019-nCoV, while also ruling out other potential complications such as tumor progression, heart failure, pulmonary embolism, and autoimmune diseases. This thorough investigation culminated in a definitive diagnosis of “grade 3 immune checkpoint inhibitor-related pneumonia.” Given the possibility of an underlying infection that could not be entirely excluded, the patient was treated with high-dose glucocorticoids alongside antibiotics, leading to a notable improvement in clinical symptoms. At this juncture, the attending physician considered the potential impact of pulmonary fibrosis on the patient’s lung function and overall quality of life following the acute phase of the illness. Drawing on a body of research regarding antifibrotic treatments, the physician devised a treatment plan that included nintedanib, which unfortunately resulted in an unexpected and rare side effect.

Nintedanib and pirfenidone, both emerging antifibrotic medications, have gained traction in the treatment of idiopathic pulmonary fibrosis (IPF). Their extensive anti-inflammatory, antioxidant, and antifibrotic properties have also yielded promising outcomes in managing non-IPF pulmonary fibrosis, demonstrating good safety and tolerability profiles. Consequently, nintedanib and pirfenidone are being explored as potential treatments for immune checkpoint inhibitor-related interstitial lung disease (ICI-ILD). Notably, nintedanib may help mitigate the risk of pneumonia associated with immune checkpoint inhibitors (ICIs) and chemotherapy (9, 10). A retrospective study indicated that combining steroids with pirfenidone is both safe and beneficial for enhancing patients’ interstitial pneumonia and reversing chemotherapy-induced pneumonitis (CIP), with ongoing use of pirfenidone potentially preventing CIP recurrence (11). However, as a vascular endothelial growth factor receptor inhibitor, nintedanib carries an increased risk of bleeding, with global pharmacovigilance data reporting a bleeding rate of 36.8 events per 1,000 patients annually (12). In a particular case, subcutaneous bleeding was observed following the initiation of nintedanib treatment, leading to further investigations that revealed thrombocytopenia and decreased fibrinogen levels. Previous research has indicated that bleeding occurrences during nintedanib therapy are not associated with thrombocytopenia (13), and the drug’s mechanism of action involving PDGFR inhibition may account for the observed decrease in platelet counts (14).

After excluding ICI-related thrombocytopenia purpura, rheumatic autoimmune diseases, infectious diseases and other blood system diseases, we used the Naranjo scale to investigate, with a score of no less than 5, indicating that the correlation between adverse reactions and medication was positive. It was concluded that the patient’s thrombocytopenia should be classified as drug-induced thrombocytopenia (DIT). Reported cases of nintedanib-induced thrombocytopenia have typically occurred one month and approximately three months after the initiation of treatment (4, 5); however, our patient experienced this condition within just one week of starting the therapy. Notably, the thrombocytopenia affected only the platelet count, with no observed impact on other blood cell lines, and it resolved rapidly within one week following the discontinuation of the medication. Therefore, we believe that immune factors may be involved in the occurrence of acute thrombocytopenia with fibrinogen consumption in this case. It is suggested that anti-platelet and fibrinogen antibodies should be detected during diagnosis and treatment to explore immune mechanisms. Currently, there is no evidence to suggest that nintedanib treatment results in fibrinogen reduction. In addition, the influence of age and renal function on pharmacokinetics should also be noted, which may also be potential risk factors for adverse reactions associated with nintedanib.

## Conclusion

In summary, we presented a case of acute thrombocytopenia and fibrinogen reduction that developed during treatment with Nintedanib. By sharing this case, we aim to increase awareness among clinicians and clinical pharmacists regarding this uncommon side effect. Given the growing use of Nintedanib for treating immune checkpoint inhibitor-related interstitial pneumonia, we will likely encounter more instances of thrombocytopenia and fibrinogen reduction in the future. Although the precise mechanism behind this thrombocytopenia remains unclear, it may be associated with PDGFR inhibition or other immune-mediated effects, highlighting the need for further research to clarify these connections. This case illustrates that thrombocytopenia and fibrinogen reduction induced by Nintedanib can arise at any point during treatment. Therefore, routine monitoring of platelet count and coagulation function in patients treated with nintedanib is strongly recommended and clinical decisions are made based on risk factors including age, liver and kidney function.

## Data Availability

The original contributions presented in the study are included in the article/Supplementary Material. Further inquiries can be directed to the corresponding author.
